# Analysis of ENSO’s response to unforced variability and anthropogenic forcing using CESM

**DOI:** 10.1038/s41598-017-18459-8

**Published:** 2017-12-22

**Authors:** Benjamin Vega-Westhoff, Ryan L. Sriver

**Affiliations:** Department of Atmospheric Sciences, University of Illinois at Urbana-Champaign, Illinois, United States of America

## Abstract

Understanding how the El Niño-Southern Oscillation (ENSO) may change with climate is a major challenge, given the internal variability of the system and relatively short observational record. Here we analyze the effect of coupled internal variability on changes in ENSO under anthropogenic global warming using the Community Earth System Model (CESM). We present results from a ~5000 year control run with constant pre-industrial conditions and a 50-member climate change ensemble experiment, consisting of historical hindcasts (1850–2005) and future projections to 2100 following representative concentration pathway 8.5 (RCP8.5). Given this large single-model ensemble, we are able to use simple statistical analyses to compare the effects of anthropogenic climate change with the effects of natural modulations in ENSO sea surface temperature (SST) metrics, as well as how internal variability may change with global warming. Changes in eastern Pacific ENSO SST metrics due to climate change are secondary to the model’s natural modulations; however, central Pacific ENSO amplitude significantly decreases, to an extent comparable with natural modulations. We also assess the sensitivity of internal variability estimates to ensemble size. The primary role of natural modulations in this ensemble highlights the importance of careful assessment of ocean-atmosphere internal variability in ENSO projections.

## Introduction

Natural (or internal) variations within Earth’s climate system are an important source of uncertainty in projections of future climate, particularly on inter-annual to decadal timescales^1^. This variability can influence historical and future projections of key climate change indicators, such as regional temperature and precipitation patterns^2^, and global temperature trends^3^. Earth system modeling approaches that capture the effect of natural variability, or more precisely the variability within the coupled system in the absence of time-varying external forcing, can be used to characterize the importance of internal variability when considering potential anthropogenic climate trends^4^. This type of approach is especially useful for ENSO, which undergoes decadal modulations that have hindered even qualitative projections of potential amplitude change in the coming decades.

**Figure 1:**
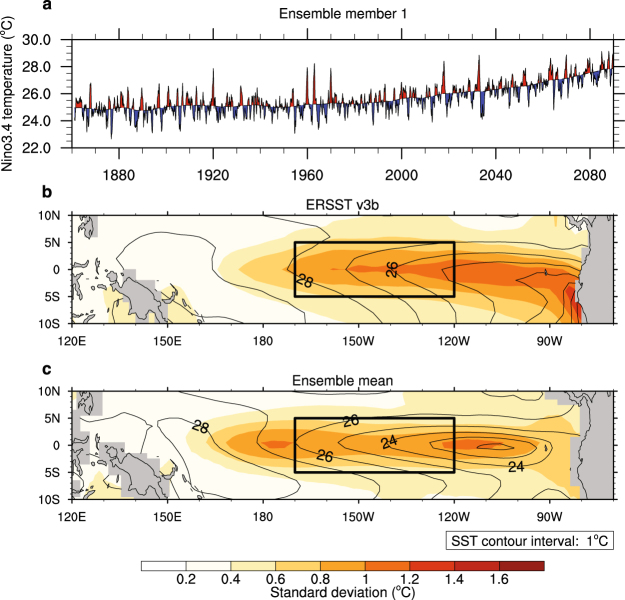
Sample Niño3.4 time series and comparison of observed and modeled mean state and SST variability. (**a**) The Niño3.4 index added to the temperature trend for a sample ensemble member. (**b**,**c**) Contours of mean SST (lined) and standard deviation of SST climatological anomalies (colored). (**b**) Reconstructed observations from 1940–1990 (ERSST v3b^52^). (**c**) The mean of the 50 CESM ensemble members from 1940–1990. In (**a**), index values above the trend are filled red, while index values below are filled blue. In (**b** and **c**) the Niño3.4 region is outlined in black. The map was generated using the NCAR Command Language (NCL), version 6.4.0 (https://www.ncl.ucar.edu).

ENSO is an important contributor to Earth’s inter-annual climate variability, with worldwide weather effects^5–7^. ENSO can be characterized with the Niño3.4 index, the climatological SST anomaly in the Niño3.4 region of the equatorial Pacific (outlined in Fig. 1). El Niño (La Niña) events correspond to a positive (negative) Niño3.4 index, with the strength of the event tied to the magnitude of the temperature anomaly (e.g., Fig. 1a). Several ENSO metrics can be derived from the Niño3.4 index time series: the spectrum reveals the frequency distribution of events, skewness roughly measures the relative strength of El Niño and La Niña events, and the standard deviation is tied to ENSO SST amplitude. In observations and simulations, ENSO events display considerable diversity, including variations in geographical location and seasonal evolution. Methods used to assess this diversity include, among others, SST indices that emphasize different location centers^8,9^. Analyses of ENSO-related feedbacks, for example the Bjerknes positive feedback between zonal winds and SST, are key to understanding ENSO mechanisms and evolution in the real world. These feedbacks also allow detailed assessment of model representations of ENSO and provide a useful framework to investigate possible ENSO changes in the future^10–12^.

In climate model simulations with constant pre-industrial conditions, ENSO frequency, event location diversity, and SST amplitude undergo strong decadal modulations. Several hundred years of data are required to sample these modulations at stable CO_2_^13–15^. In the most recent multi-model ensemble, the Coupled Model Intercomparison Project (Phase 5) (CMIP5)^16^, there is no consensus change in ENSO SST metrics under global warming^17,18^, and if there are robust changes, they may be time-dependent^19^. However, the ensemble does display robust changes in other key ENSO properties, such as the associated precipitation anomalies and event propagation characteristics^18,20,21^.

Here, rather than using a multi-model approach that samples structural model uncertainty in the processes that govern ENSO, we analyze a large ensemble from a single model that performs reasonably well in simulating realistic ENSO-like behavior (Fig. 1). The ensemble samples the internal variability of the coupled ocean-atmosphere-land-sea ice system, which we define as its year-to-year variations in the absence of time-varying greenhouse gas forcing. Our ensemble uses a low-resolution configuration of CESM (T31 × 3, described in the Methods section). Fifty individual ensemble members branch off at 100-year intervals from a fully-coupled, equilibrated, pre-industrial control simulation. Each member is then forced with historical values through 2005, then RCP8.5 until 2100. Because each ensemble member is identically forced after being initialized from its own unique starting point (corresponding to different times) in the equilibrated control, any differences between members are solely due to internal variability of the coupled climate system^22,23^. This single-model approach, including both control and forced data, allows for comparisons of ENSO’s internal variability and response to climate change. The ensemble size allows for straight-forward and robust statistical assessment including quantile analysis, unlike smaller single-model assessments that use statistical estimators requiring key assumptions about stationarity or representativeness of a relatively short dataset (given ENSO’s decadal modulations)^17,24,25^.

While this configuration of low-resolution CESM exhibits mean state biases in equatorial Pacific SST, analysis of a similar configuration of CCSM4 and our own investigation show that it captures realistic variability associated with ENSO^26^ (Fig. 1). In this regard it is comparable with members of the CMIP5^27^. This configuration of low-resolution CESM roughly reproduces tropical Pacific mean state seasonality, as well as ENSO’s seasonal phase locking (Supplementary Figs S1 and S2). The model also simulates the recharge-discharge mechanism of the equatorial Pacific, in which changes in ocean heat content accumulate before ENSO events occur^28,29^ (Supplementary Fig. S3).

**Figure 2:**
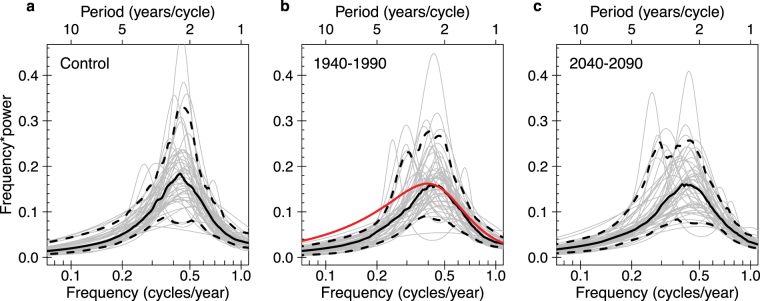
Maximum entropy power spectra of the trend-removed Niño3.4 index under different forcing regimes. (**a**) Fifty 50-year sections of the unforced, control CESM simulation; (**b**) the 50 CESM ensemble members (1940–1990) and, in red, the 1940–1990 reconstructed observational data (ERSST v3b); (**c**) the 50 CESM ensemble members (2040–2090). Individual members are shown in grey, dashed curves are the 5^th^ and 95^th^ percentiles, and the solid black curve is the median.

We examine ENSO in three different forcing regimes: constant pre-industrial forcing, 1940–1990 historical forcing, and RCP8.5 2040–2090 projected forcing. Each forcing regime ensemble has 50 independent members sampling ENSO’s natural modulations. Figure 2 shows the ENSO maximum entropy spectra for each forcing regime. Each ensemble exhibits considerable variety of spectral shape, with some members peaking around four years and others at less than two years, while the normalized power in those peaks can vary by greater than a factor of four. At the ensemble peak frequency, the power for individual members ranges from ~1/3 the median power to a factor of 2.5 greater than the median. The medians of each forcing regime ensemble, however, are similar in shape. While there are noticeable differences in the 95% confidence intervals of the ensemble means (e.g., a smaller peak in 2040–2090), the intervals overlap for much of the range (Supplementary Fig. S4). These basic results are consistent using a fast Fourier transform (Supplementary Figs S5 and S6).

**Figure 3:**
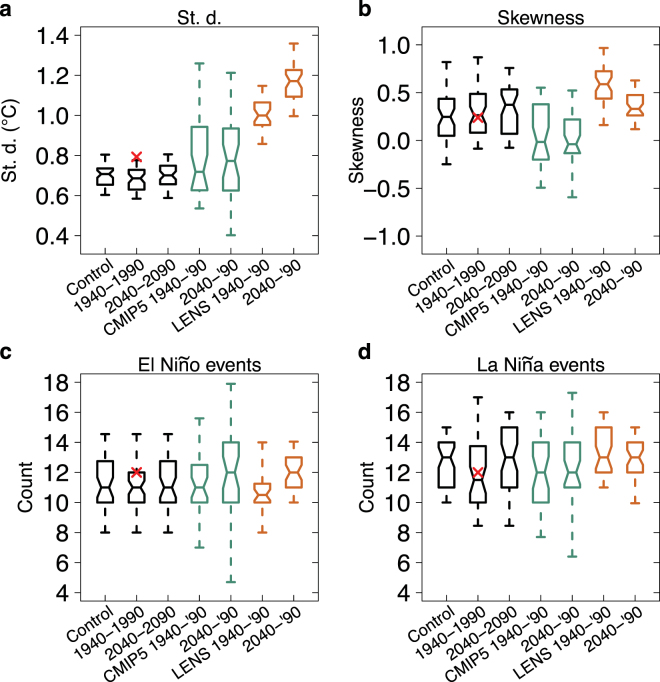
Box-whisker plots of ENSO statistics under different forcing regimes. (**a**) Trend-removed Niño3.4 index standard deviation; (**b**) skewness; (**c**) El Niño event counts. (**d**) La Niña event counts. The plots show 5^th^, 25^th^, 50^th^, 75^th^, and 95^th^ percentiles for fifty 50-year sections of the unforced, control CESM run (Control), the 50 CESM ensemble members from 1940–1990 (1940–1990), and the 50 CESM ensemble members from 2040–2090 (2040–2090). In green are the distributions for the 35-member CMIP5 ensemble. In orange are the distributions for the 40-member LENS ensemble. The red x denotes the 1940–1990 reconstructed observational value (ERSST v3b). The notches indicate a rough 95% confidence interval for the difference between two medians.

Figure 3 compares the distributions of several statistical properties of the Niño3.4 index in the different forcing regime ensembles. Standard deviation, an indicator of ENSO SST amplitude, is somewhat underestimated in the model. The observed 1940–1990 value is above the 95^th^ percentile of the 1940–1990 ensemble. The distributions for each forcing regime have similar widths and sizeable overlap. CMIP5 and CESM Large Ensemble (LENS; discussion of the CESM ensemble below specifically refers to our low-resolution ensemble unless otherwise noted)^30^ distributions are also shown. CMIP5 distributions, sampling a mix of internal variability and model uncertainty, are wider than those of our CESM ensemble (Fig. 3a). LENS, run at a higher resolution than our ensemble and sampling only different atmospheric initial conditions, exhibits distributions that are similar in width to those from our ensemble, though there is a noticeable increase in standard deviation under projected forcings.

Skewness roughly indicates the relative strength of El Niño and La Niña events. The observed 1940–1990 skewness is positive (El Niño events are generally stronger than La Niña), and closely matches the CESM ensemble median over the same time period. Similar to standard deviation, there is sizeable overlap between the CESM forcing regime ensembles (Fig. 3b). The CESM ensemble distributions are again comparable for El Niño and La Niña event counts (as defined in the Methods section) (Fig. 3c,d).

Given approximate normality (see Q-Q plots in Supplementary Fig. S7), we perform two-sided t-tests comparing the pre-industrial ensemble mean with that of the 2040–2090 ensemble mean for each statistical property. None of the differences are significant at 95% confidence, even given adequate detection sensitivity allowed by this large dataset (e.g., we could have rejected the hypothesis of equal means for a change in standard deviation greater than ~4%) (Supplementary Table S1).

**Figure 4:**
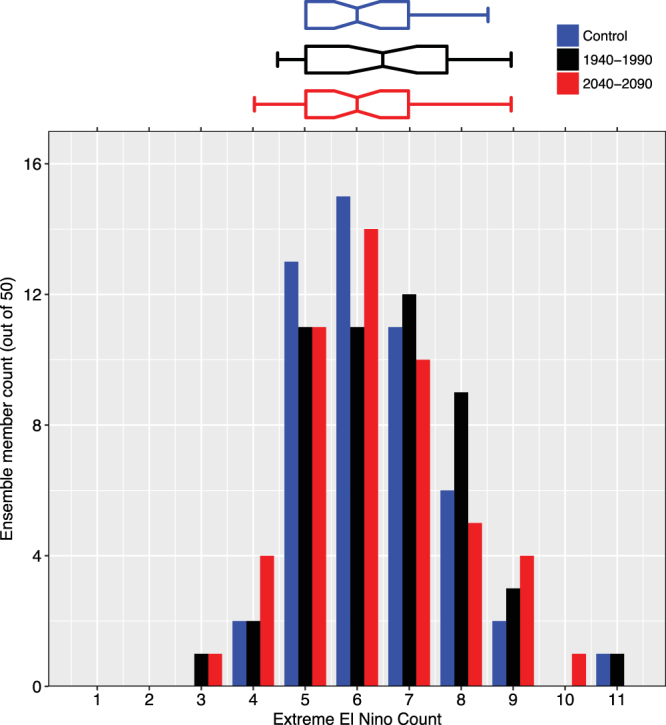
Histograms of extreme El Niño counts in different forcing regime ensembles. Fifty 50-year sections of the unforced, control CESM simulation (blue), the 50 CESM ensemble members from 1940–1990 (black), the 50 CESM ensemble members from 2040–2090 (red). Extreme El Niño events defined here as trend-removed Niño3.4 index greater than 1.75 standard deviations above mean. For reference, the count of extreme El Niño events in reconstructed observations (ERSST v3b) from 1940–1990 using this calculation is 6. The box-whisker plot above shows 5^th^, 25^th^, 50^th^, 75^th^, and 95^th^ percentiles. Notches indicate a rough 95% confidence interval for the difference between two medians.

Extreme El Niño has different dynamics from moderate events and therefore may be differently affected by climate change^18^. In CMIP5, there is inter-model consensus for a projected increase in extreme event frequency using precipitation-based metrics. However, there is no such consensus using temperature-based metrics. Continuing our temperature-based analysis, we adapt a published SST-based identification^31^ (described in the Methods section) to examine extreme ENSO in the CESM ensemble (Fig. 4). Comparing extreme El Niño counts in different forcing regimes, we note that these discrete counts are approximately normal (Supplementary Fig. S7) and perform two-sided t-tests. We find no significant difference in mean event counts (p-value = 0.84 comparing control and 2040–2090, see Supplementary Table S1). We also note a large range within each regime ensemble, with some members having only three extreme events in 50 years, while others have 11.

**Figure 5:**
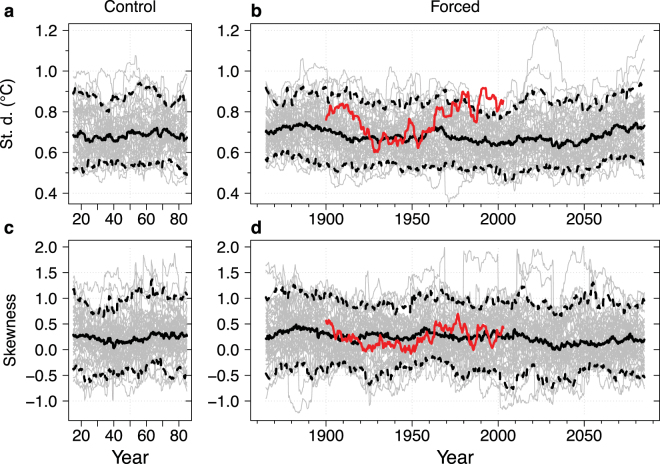
Running ENSO statistics in the CESM ensemble. (**a**,**b**) 20-year running standard deviation of the trend-removed Niño3.4 index; (**c**,**d**) skewness. (**a**,**c**) 50 sections of the unforced, control CESM run; (**b**,**d**), the 50 CESM ensemble members. Individual members are shown in grey, dashed lines are the 5^th^ and 95^th^ percentiles, the solid black line is the median, and the solid red line is the reconstructed observational record (ERSST v3b).

For an explicit analysis of ENSO evolution, we also examine the continuous record of 20-year running ENSO statistical properties. Individual simulations under both pre-industrial and climate change forcings exhibit ENSO SST amplitude (i.e., Niño3.4 index standard deviation) modulations comparable with those in the observations, and the spread due to these modulations is roughly constant through time. Modulations of ENSO skewness larger than those in the observations occur under both pre-industrial and climate change forcings. Over the combined historical and projection time period, any ensemble change in either statistical property is negligible compared with natural modulations (Fig. 5).

There can also be different types or ‘flavors’ of ENSO: the eastern Pacific type, in which SST anomalies are centered in the east, and the central Pacific type, in which SST anomalies occur near the International Date Line^8,32^. Because their underlying dynamics may be different^33,34^, these events may respond differently to climate change^35^. CMIP5 ensemble projections show either an increase or no significant change in central Pacific ENSO amplitude, depending on methodology. Some assessments of CMIP5 subsets project increased central Pacific ENSO frequency or amplitude^20,35^, while others indicate no robust changes^36,37^. Analyses of the overall CMIP5 ensemble show no significant change in central Pacific ENSO indices^20,36^.

We repeat the CESM ensemble analysis of running statistical properties for two additional regions: Niño3 (5°S-5°N, 150°W-90°W), which is more strongly affected by eastern Pacific events, and Niño4 (5°S-5°N, 160°E-150°W), more strongly affected by central Pacific events^34^. Like Niño3.4, these regions exhibit natural modulations larger than any ensemble changes (Supplementary Figs S8 and S9). However, unlike Niño3.4 and Niño3, the ensemble Niño4 amplitude is significantly lower in 2040–2090 compared to pre-industrial (5% lower with two-sided t-test p-value = 0.002).

**Figure 6:**
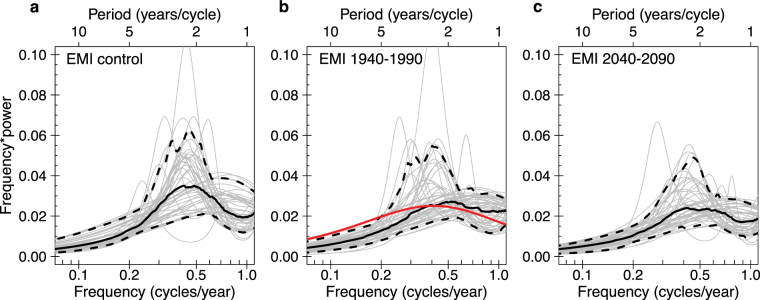
Maximum entropy power spectra of the El Niño Modoki index under different forcing regimes. (**a**) Fifty 50-year sections of the unforced, control CESM simulation; (**b**) the 50 CESM ensemble members (1940–1990) and, in red, the 1940–1990 reconstructed observational data (ERSST v3b); (**c**) the 50 CESM ensemble members (2040–2090). Individual members are shown in grey, dashed curves are the 5^th^ and 95^th^ percentiles, and the solid black curve is the median.

To investigate the possible change in central Pacific ENSO, we repeat analyses using the El Niño Modoki Index (EMI), an index for central Pacific ENSO that is less affected by eastern Pacific events than the Niño4 index (defined in Methods)^8^. There is a more significant decrease in EMI standard deviation from pre-industrial to 2040–2090, with two-sided t-test p-value = 2.7*10^−12^ (Supplementary Table S1). There is a corresponding decrease in spectral power (Fig. 6, Supplementary Fig. S4). We therefore conclude that the ensemble’s central Pacific ENSO SST amplitude significantly decreases with increased anthropogenic forcing. This decrease contrasts with both the observed upward trend (Supplementary Fig. S9) and general CMIP5 projections, though central Pacific ENSO amplitude does apparently decrease in a couple of the small single-model ensembles provided for CMIP5^36^.

The different responses to global warming in east and central Pacific ENSO may be related to a different balance of compensating effects in the two regions. To explore the existence of compensation, two key ENSO-related feedbacks, the negative heat flux and positive Bjerknes feedbacks, are analyzed in historical and projection time periods. The two feedbacks can be quantified as the slope of a linear regression of surface temperature anomalies with heat flux anomalies^38^ and Niño4 zonal wind stress anomalies^39^, respectively. We find an enhancement of the heat flux feedback and negligible change in the Bjerknes feedback in future projections (Supplementary Fig. S10). Changes in other feedbacks likely compensate for the enhanced negative heat flux feedback, such as positive thermocline feedback due to future shoaling of the equatorial Pacific thermocline^11,40^. An expected weakening of the negative feedback due to east Pacific mean upwelling and subsurface advection in the future^11,41^, associated with preferential warming in the east equatorial Pacific (Supplementary Fig. S11), may also offset the enhanced negative heat flux feedback and help explain the lack of an apparent significant change in the Niño3.4 index.

The lack of an ensemble change in Niño3.4 index contrasts with results from related models: the Community Climate System Model v4 (CCSM4) projection from CMIP5^36,42,43^ and LENS ensemble results (Fig. 3)^44^. The single run of CCSM4 in CMIP5 undergoes a decrease in amplitude that is much larger than our estimated internal variability, and the decrease occurs across the Niño3 and Niño4 regions^36^. Analysis of this decrease and changes in other CMIP5 models shows that ENSO’s response to global warming may be tied to the mean state response of eastern equatorial Pacific meridional flow^42,45^. Changes in mean state meridional flow affect ENSO meridional structure, with larger mean poleward motion associated with a wider ENSO structure and lower amplitude. Indeed, the CCSM4 meridional ENSO structure widens in projections, while high-resolution CESM becomes slightly narrower, and our CESM ensemble ENSO structure undergoes a relatively slight widening (Supplementary Fig. S12)^45^. The increased ENSO amplitude in LENS could be related to changes in the mean meridional flow, however, it may also be related to the ensemble set-up. Unlike our ensemble, which samples unique initial conditions of the fully-coupled climate system, each member of LENS is initialized with the same ocean state in 1920, differentiated only by atmospheric perturbations. Therefore, the ensemble does not fully sample ocean internal variability. The significant ENSO amplitude increases in the early decades of LENS may be related to this issue^44^, which may persist for 50 years or longer based on the adjustment time scale of the low-resolution CESM ocean^23^. Model resolution may also play a key role in ENSO’s response to global warming, and has been shown to affect ENSO-related feedbacks, such as the Bjerknes feedback^46^.

**Figure 7:**
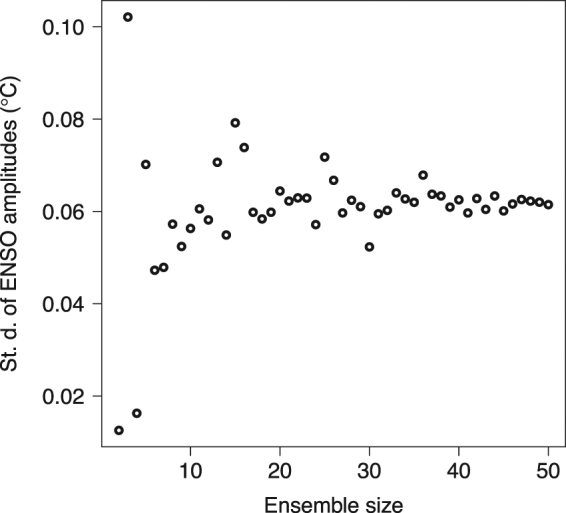
Estimated internal variability of 50-year ENSO amplitude versus ensemble size. The internal variability estimate is calculated as the standard deviation of the ensemble distribution of 50-year Niño3.4 index standard deviations (ENSO amplitudes). Ensembles are created by sampling 50-year sections of the unforced, control CESM simulation, without replacement.

A large single-model forced ensemble allows both high-sensitivity investigation of ENSO’s changes due to forcing, as well as robust estimates of ENSO internal variability. As earlier noted, analyses of long control simulations have shown that several hundred years are required to sufficiently sample ENSO modulations under a given forcing^13,14^. We pose a related question: how many ensemble members are necessary to provide adequate sampling of internal variability of ENSO 50-year metrics? Given approximate normality of the ensemble distribution of a 50-year metric (Supplementary Fig. S7), its internal variability can be described by the standard deviation of that distribution. Then, deciding the appropriate size of an ensemble to assess natural modulations is essentially deciding the appropriate number of samples from a normal distribution to assess its standard deviation. Sampling without replacement from our 50 control ensemble members, we calculate the sample standard deviation of ENSO amplitude (i.e., Niño3.4 index standard deviation) as a function of ensemble size (Fig. 7). Ensembles with less than ~20 members will poorly assess internal variability of 50-year averaged metrics. Using the full ensemble, internal variability estimates for all three forcing regimes (0.061 °C, 0.067 °C, and 0.070 °C for control, 1940–1990, and 2040–2090, respectively) are much larger than the magnitude of the ensemble mean amplitude change (−0.003 °C from control to 2040–2090). The internal variability of 50-year EMI standard deviation is roughly constant in all three forcing regimes (0.026 °C, 0.027 °C, and 0.026 °C for control, 1940–1990, and 2040–2090, respectively) and comparable with, though less than, the ensemble mean decrease (0.041 °C from control to 2040–2090, Supplementary Table S1).

In summary, we use an ensemble framework that allows explicit estimation and comparison of ENSO’s coupled internal variability and response to different forcing regimes. In this CESM ensemble, we find that the effect of climate change on model eastern Pacific ENSO SST is secondary to natural modulations, while central Pacific ENSO amplitude significantly decreases, to an extent comparable with natural modulations. The primary role of internal variability here highlights its importance to investigations of ENSO’s climate change response. Earth system model ensembles such as this one, that adequately sample the coupled ocean-atmosphere internal variability, can be used to estimate the model ENSO’s internal variability and its potential non-stationarity. This approach enables a robust assessment of the modeled ENSO’s response to radiative forcing, as well as its detectability in the presence of internal variability.

## Methods

### CESM experiment

The experiment uses the fully-coupled low-resolution configuration of the Community Earth System Model (CESM), T31 × 3^22,23^. The CAM4 atmospheric model component is configured with a spectral resolution of ~3.75° × 3.75° and 26 vertical levels. The ocean model component has a nominal horizontal resolution of 3° (changing to less than 1° near the equator) and 60 vertical levels. While this configuration of low-resolution CESM has several climate biases, analysis of a similar configuration of CCSM4 along with our own analysis show that it captures tropical Pacific inter-annual temperature variability associated with ENSO and other properties related to ENSO^26^ (Fig. 1 and Supplementary Figs S1–S3).

We first completed a ~4000-year equilibration simulation with pre-industrial forcings, allowing the deep ocean to reach near-dynamic equilibrium. After equilibration, the control simulation continues for another 5000 years. We initialize 50 different climate change simulations from the equilibrium pre-industrial control at 100-year intervals after the 4000-year spin-up. Each simulation is initiated from its own unique snapshot of the coupled atmosphere-ocean-land-sea ice system. These 50 simulations run from 1850–2100, using historical anthropogenic and natural forcings between 1850 and 2005 and RCP8.5 from 2006 to 2100^47^. The 50-member ensemble, with identical forcings, only samples unique initialization times in the equilibrated control^48^. Thus, differences between members reflect uncertainty due to joint internal unforced variability of the fully-coupled system (ocean, atmosphere, land, and sea ice components).

We further break up the ensemble into three different analysis periods: 1940–1990 representing current climate conditions, 2040–2090 representing future climate conditions (corresponding to RCP8.5), and 50 different 50-year time slices sampled from the pre-industrial control simulation. Thus, we have three ensembles that sample the internal variability in three different forcing regimes. Repeating the analysis with longer time sections did not change our results (not shown).

### Statistical Analysis

We focus on SST in the Niño3.4 region, (5°S-5°N, 170°-120°W). We calculate a Niño3.4 index as the monthly average SST climatological anomaly in the region. To isolate ENSO variability from long-term warming, a 211-month triangle smoothing is subtracted from the index time series (as in Fig. 1 of Wittenberg, 2009). The resulting trend-removed Niño3.4 index time series has no warming trend. There are differences in the trend time series between ensemble members, meaning that some amount of internal variability is lost with this method. However, the differences in the trends are about an order of magnitude smaller than the differences between trend-removed indices (Supplementary Fig. S13).

For the spectral analysis, we calculate the maximum entropy power spectrum^49^ for each trend-removed time series. In order to visually isolate the ENSO peak, the spectrum is normalized by frequency. With the maximum entropy technique, the shape of the spectrum is sensitive to the order of the autoregressive fit, as determined using the Akaike Information Criterion. For example, maximum entropy spectra for ERSST v3b and HadISST v1.1^50^ are notably different, even though they are produced from almost identical index time series (Supplementary Fig. S14). We repeat our spectral analysis using a fast Fourier transform, which involves no smoothing of the time series, and basic results are unaffected (Supplementary Figs S5 and S6).

For box-and-whisker plots, the notches extend to ±(1.58 * *IQR*)$/\sqrt{n}$ where *IQR* is the interquartile range and *n* is the number of ensemble members (50 for our CESM ensemble, 35 for CMIP5, and 40 for LENS). The notches give a rough 95% confidence interval for the difference between two medians^51^. El Niño (La Niña) events are identified by at least 5 consecutive months in which the three-month average of the trend-removed index is greater (less) than 0.5 °C (−0.5 °C), similar to the method used by NOAA Climate Prediction Center. CMIP5 distributions are determined from a 35-member ensemble. The full list is provided in Supplementary Table S2.

Extreme El Niño events are identified by a trend-removed Niño3.4 index that is higher than 1.75 standard deviations above the mean and not following within five months of another extreme event. This definition is adapted from a published Niño3-based definition^31^.

The El Niño Modoki index (EMI) is calculated as follows:

1 $${\rm{EMI}}={[{\rm{SSTA}}]}_{{\rm{A}}}-0.5\ast {[{\rm{SSTA}}]}_{{\rm{B}}}-0.5\ast {[{\rm{SSTA}}]}_{{\rm{C}}},$$

with brackets indicating area-averaged climatological SST anomalies over regions A (165°E-140°W, 10°S-10°N), B (110°W-70°W, 15°S-5°N), and C (125°E-145°E, 10°S-20°N), respectively^8^.

### Data Availability

The CESM ensemble raw ENSO regional time series are available in text format as supplementary data. Other datasets and codes used during the current study are available from the corresponding author on reasonable request.

## Electronic supplementary material

Supplementary Info
