# Supplementary material for: Analysis of ENSO’s response to unforced variability and anthropogenic forcing using CESM
Source: Sci Rep. 2017 Dec 22;7:18047. doi: 10.1038/s41598-017-18459-8 (PMC5741761; doi:10.1038/s41598-017-18459-8)
Supplement: Supplementary file 1 — Supplementary Info [file 41598_2017_18459_MOESM1_ESM.pdf]

Analysis of ENSO's response to unforced variability and anthropogenic forcing using CESM

Benjamin Vega-Westhoff, Ryan L. Sriver*

Department of Atmospheric Sciences, University of Illinois at Urbana-Champaign, Illinois, United States of America. Correspondence and requests for materials should be addressed to R.L.S. (email: rsriver@illinois.edu)

Supplementary information

Supplementary figures

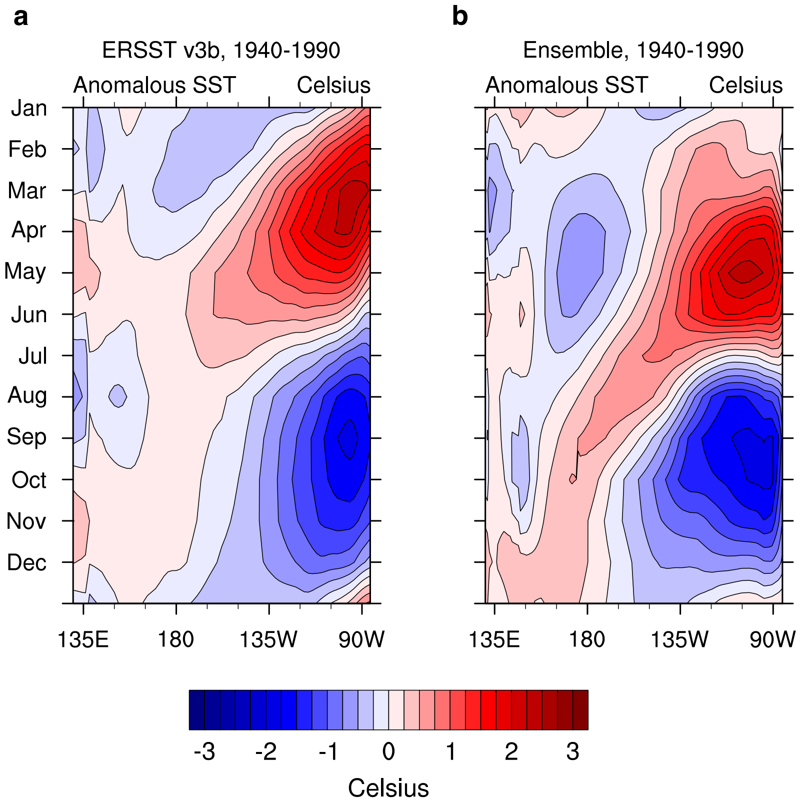

Supplementary Figure S1 | Annual cycle of equatorial Pacific surface temperature in observations and the CESM ensemble. Longitude-time plots of the annual cycle of surface temperature anomalies in the equatorial Pacific (5°S–5°N) relative to the annual meridional mean from 1940-1990 for **a**, reconstructed observations (ERSST v3b) and **b**, the CESM ensemble average.

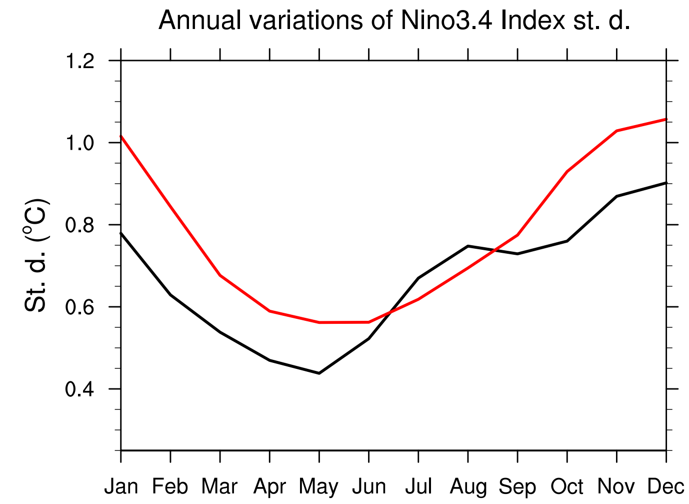

Supplementary Figure S2 | ENSO phase locking in observations and the CESM ensemble. Monthly average standard deviation of trend-removed Niño3.4 index from 1940-1990 for reconstructed observations (red), and the CESM ensemble average (black**).**

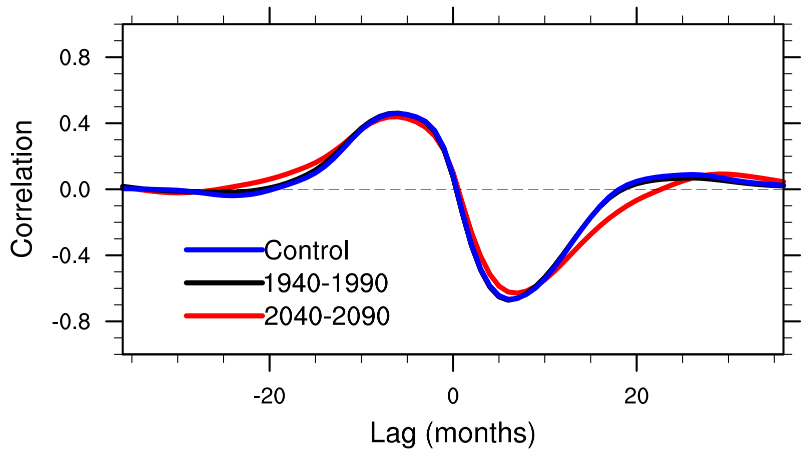

Supplementary Figure S3 | Lagged cross-correlation of trend-removed Niño3.4 (5°S-5°N, 170°W-120°W) index and 20°C isotherm depth averaged over the equatorial Pacific (5°S-5°N, 120°E-90°W). These are ensemble averages over fifty 50-year sections of the unforced, control CESM simulation (blue), the 50 CESM ensemble members from 1940-1990 (black), and the 50 CESM ensemble members from 2040-2090 (red). Negative lags indicate that 20° isotherm depth anomalies lead Niño3.4 SST anomalies.

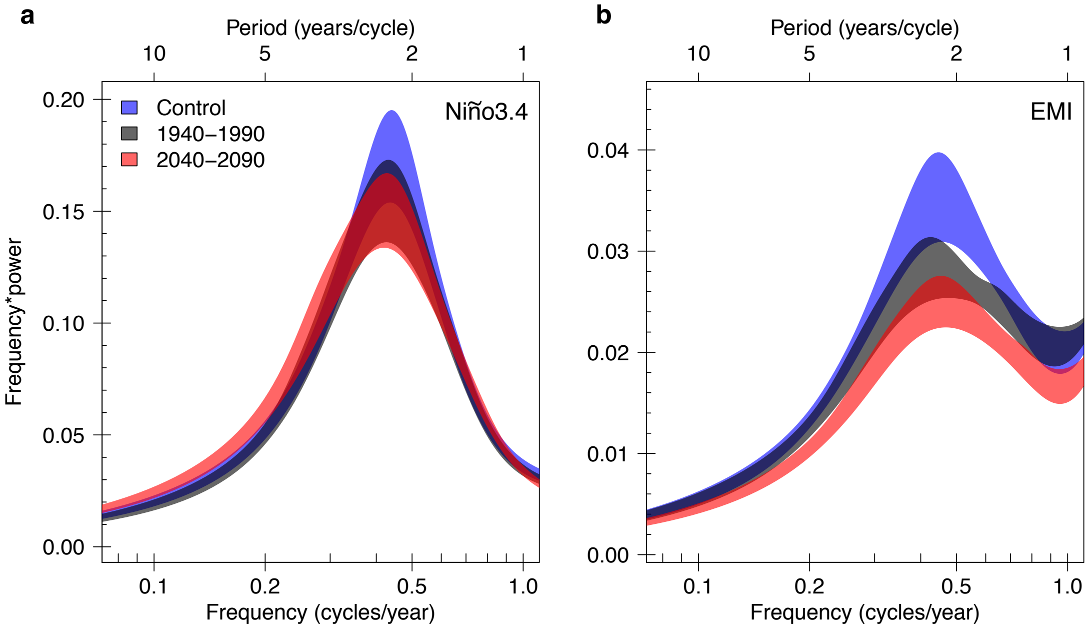

Supplementary Figure S4 | 95% confidence intervals for the mean maximum entropy power spectrum under different forcing regimes. **a**, The trend-removed Niño3.4 index; **b**, the El Niño Modoki index. Fifty 50-year sections of the unforced, control CESM simulation (blue), the 50 CESM ensemble members from 1940-1990 (black), the 50 CESM ensemble members from 2040-2090 (red).

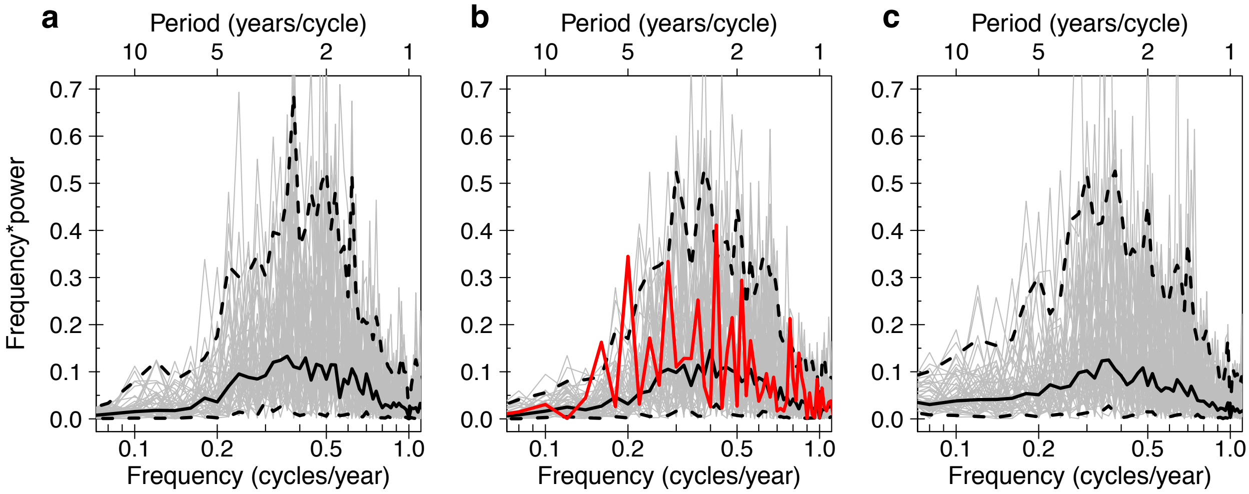

Supplementary Figure S5 | Fast Fourier transform power spectra of the trend-removed Niño3.4 index under different forcing regimes. a, Fifty 50-year sections of the unforced, control CESM simulation; **b**, the 50 CESM ensemble members (1940-1990) and, in red, the 1940-1990 reconstructed observational data (ERSST v3b); **c**, the 50 CESM ensemble members (2040-2090). Individual members are shown in grey, dashed curves are the 5th and 95th percentiles, and the solid black curve is the median.

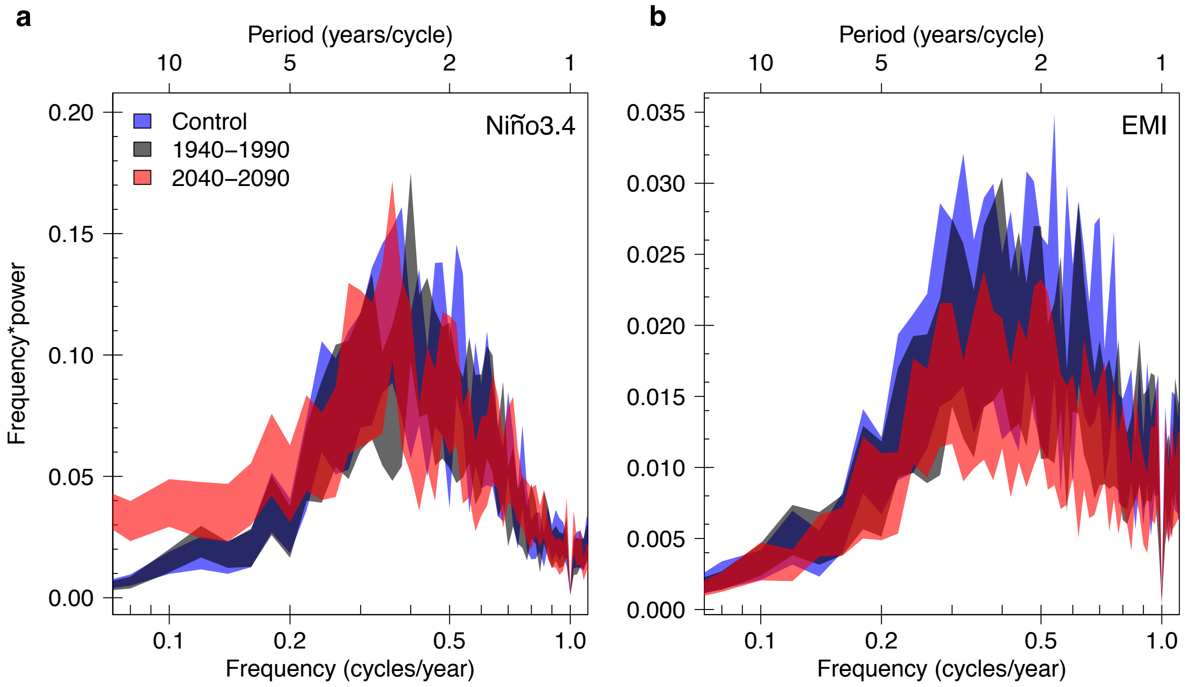

Supplementary Figure S6 | 95% confidence intervals for the mean fast Fourier transform power spectrum under different forcing regimes. **a**, The trend-removed Niño3.4 index; **b**, the El Niño Modoki index. Fifty 50-year sections of the unforced, control CESM simulation (blue), the 50 CESM ensemble members from 1940-1990 (black), the 50 CESM ensemble members from 2040-2090 (red).

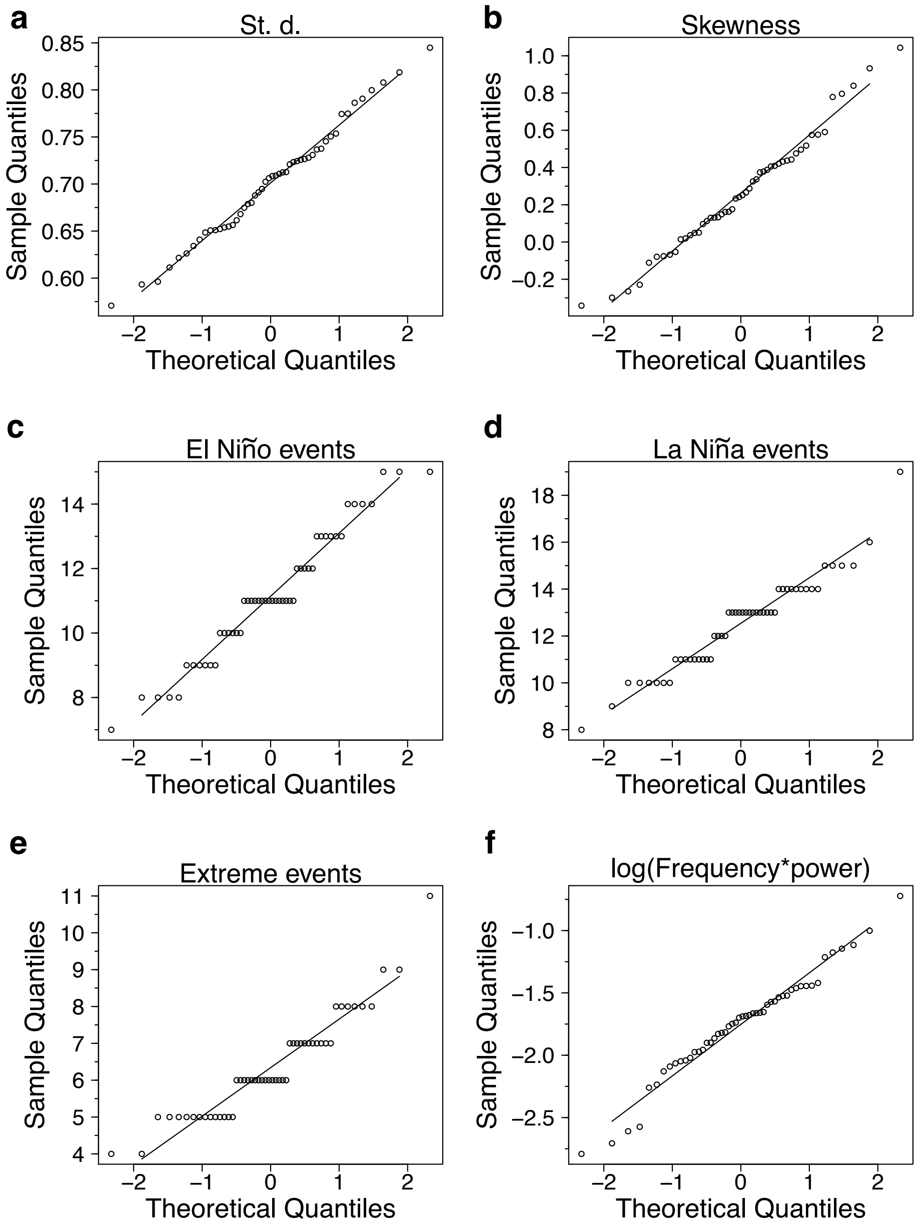

Supplementary Figure S7 | Quantile-quantile plots comparing control ensemble distributions on the y-axis with normal distributions on the x-axis. a, Niño3.4 standard deviation; **b**, skewness; **c**, El Niño event counts; **d**, La Niña event counts; **e**, extreme El Niño event counts; **f**, natural log of normalized spectral power at its peak frequency. If a distribution were perfectly normal, its quantile-quantile plot would be perfectly linear.

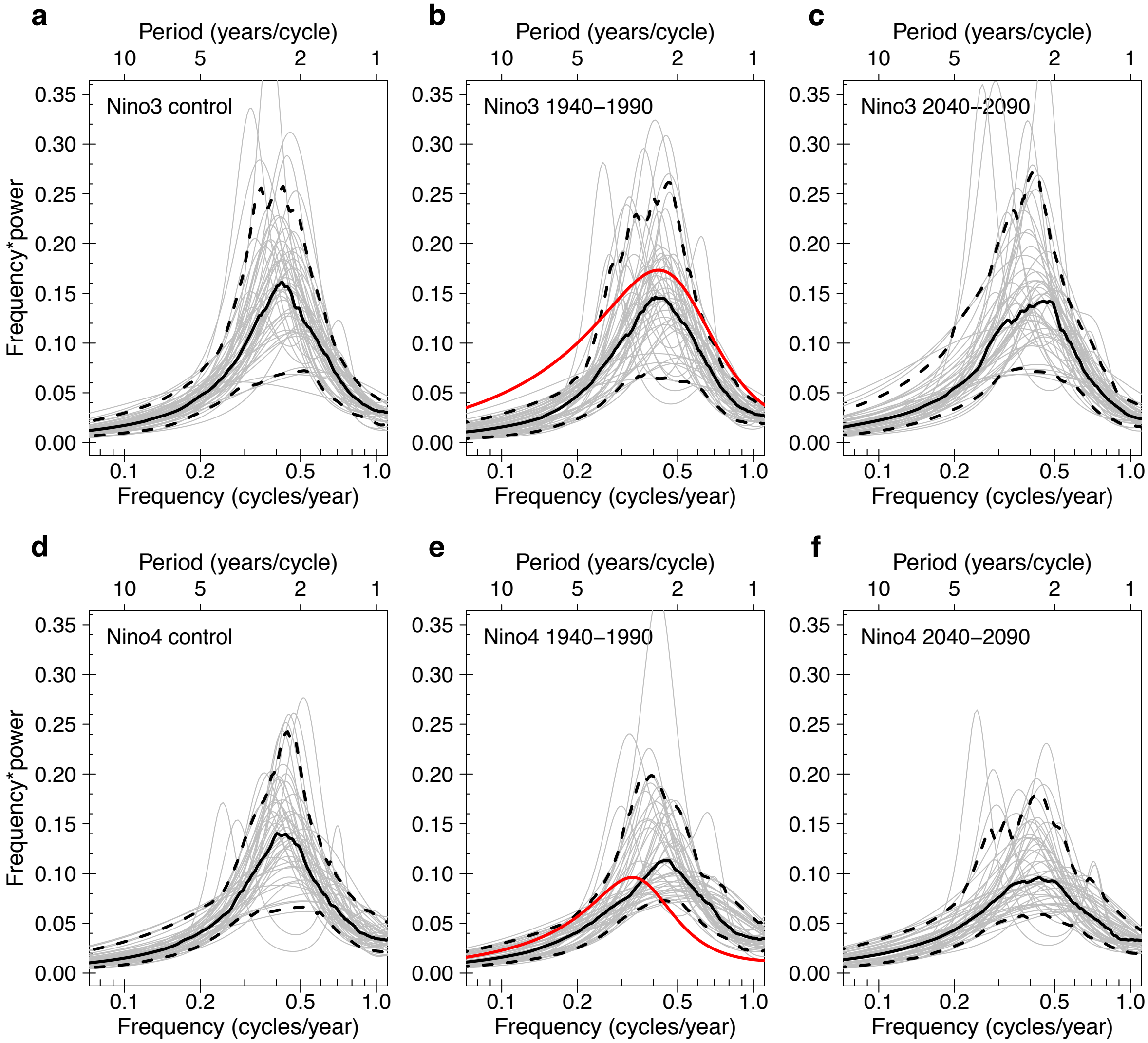

Supplementary Figure S8 | Maximum entropy power spectra for different ENSO indices and forcing regimes. **a**, **b**, **c**, Trend-removed Niño3 index in different forcing regimes; **d**, **e**, **f**, Trend-removed Niño4 index in different forcing regimes. **a**, **d**, Fifty 50-year sections of the unforced, control CESM simulation; **b**, **e**, the 50 CESM ensemble members (1940-1990); **c**, **f**, the 50 CESM ensemble members (2040-2090). Individual members are shown in grey, dashed curves are the 5th and 95th percentiles, and the solid black curve is the median.

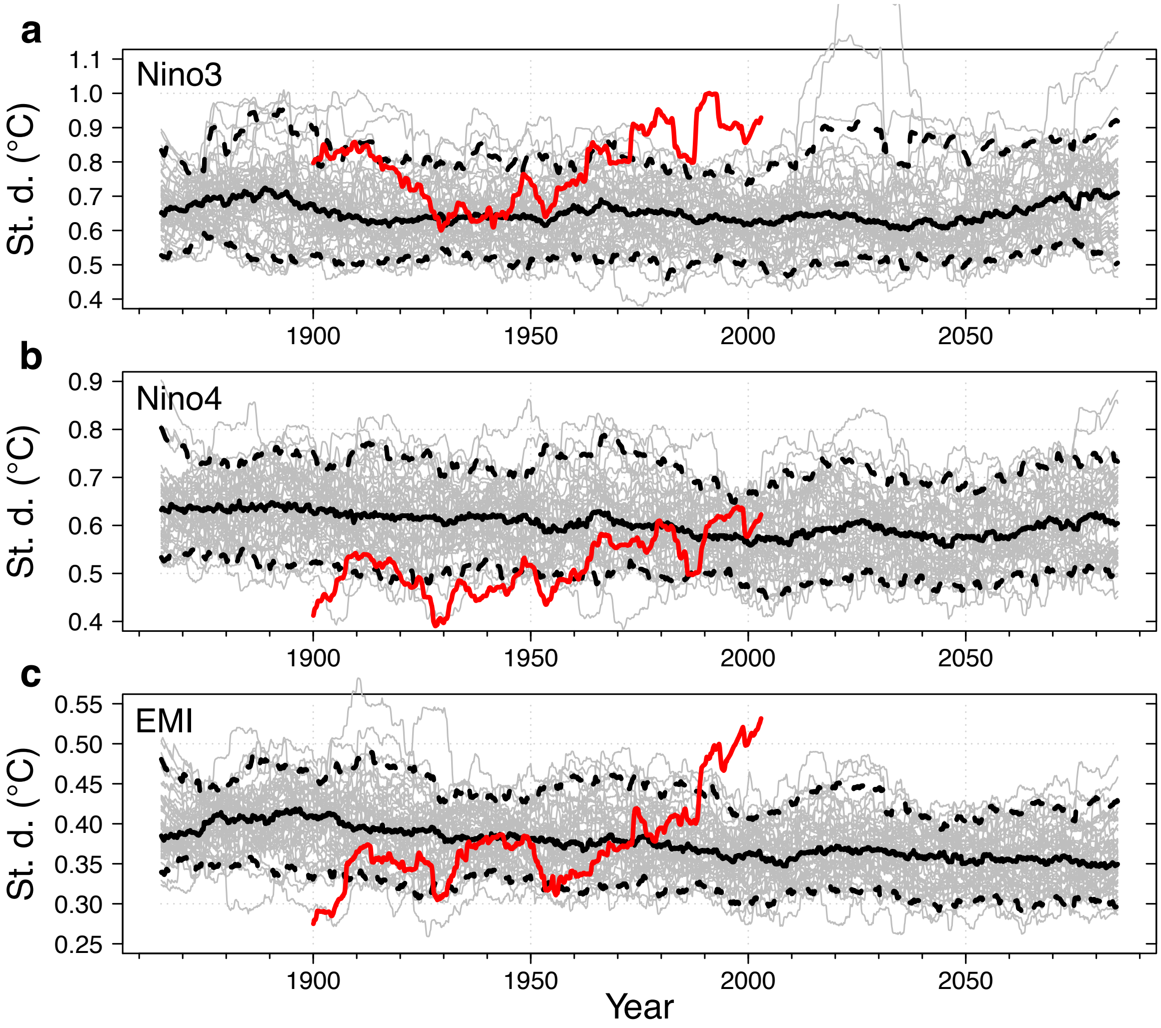

Supplementary Figure S9 | 20-year running standard deviation for different ENSO indices. **a**, Trend-removed Niño3 index; **b**, trend-removed Niño4 index; **c**, El Nino Modoki index, combining trend-removed temperature from three tropical Pacific areas. The 50 CESM ensemble members are shown in grey, dashed lines are the 5th and 95th percentiles, and the solid black line is the median.

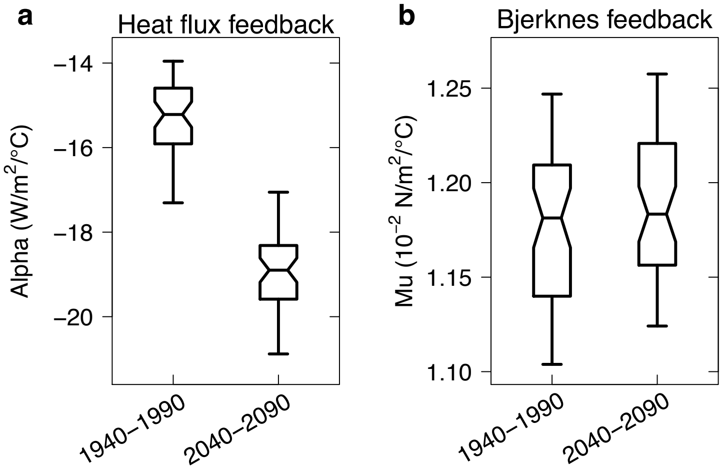

Supplementary Figure S10 | ENSO-related feedbacks in different forcing regime ensembles. a, Heat flux feedback, calculated as the slope of a linear regression between the trend-removed Niño3.4 index and anomalous heat flux in Niño3.4. **b**, Bjerknes feedback, calculated as the slope of a linear regression between the trend-removed Niño3.4 index and Niño4 anomalous zonal wind stress. Plots show 5th, 25th, 50th, 75th, and 95th percentiles for the 50 CESM ensemble members from 1940-1990 (1940-1990), and the 50 CESM ensemble members from 2040-2090 (2040-2090). The notches indicate a rough 95% confidence interval for the difference between two medians.

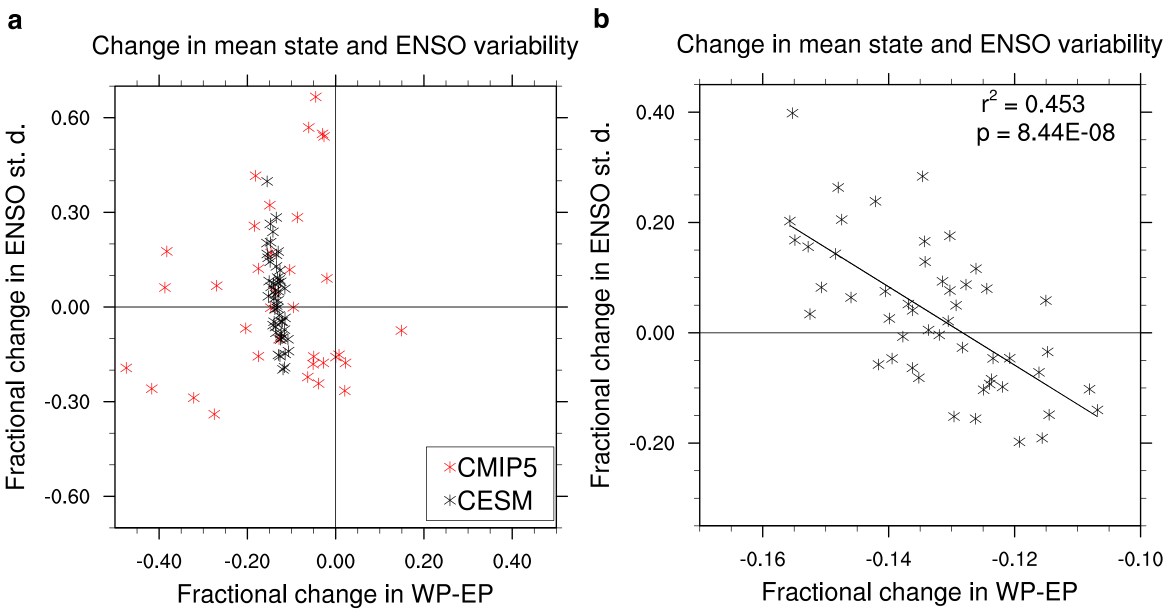

Supplementary Figure S11 | Changes in the equatorial zonal temperature gradient and Niño3.4 standard deviation. a, Fractional change in western equatorial Pacific (5°S-5°N, 120°E-170°E) minus eastern equatorial Pacific (5°S-5°N, 150°W-90°W) SST vs. fractional change in trend-removed Niño3.4 standard deviation from 1940-1990 to 2040-2090. In red are the 35 CMIP5 ensemble members; in black are the 50 CESM ensemble members. Negative fractional changes in WP-EP indicate preferential warming in the east. b, Same as a, but only showing the CESM ensemble members. The listed p-value is that of a two-sided t-test of the null hypothesis of zero slope.

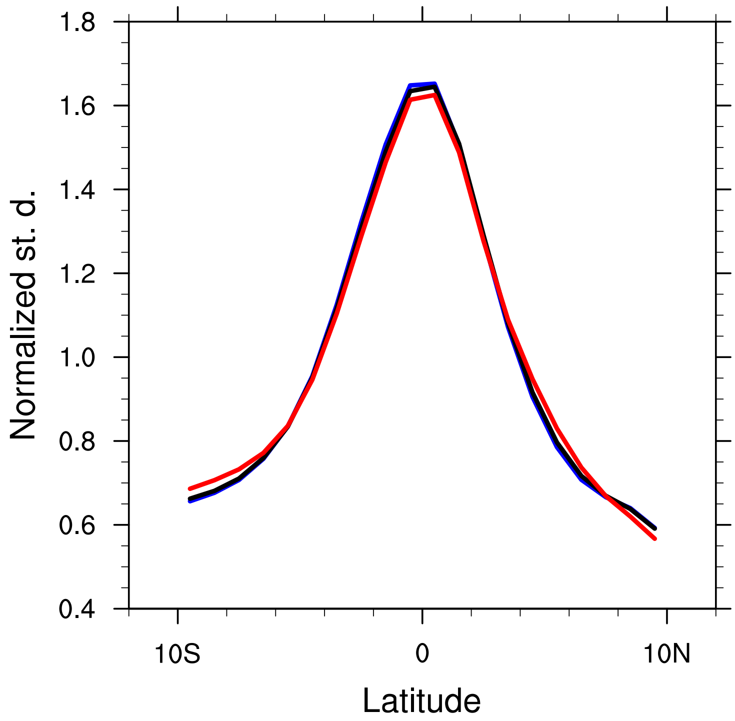

Supplementary Figure S12 | ENSO meridional extent in different forcing regimes. Meridional profile of the normalized standard deviation of trend-removed eastern Pacific (150°W-90°W) temperature anomalies. These are ensemble averages over fifty 50-year sections of the unforced, control CESM simulation (blue), the 50 CESM ensemble members from 1940-1990 (black), and the 50 CESM ensemble members from 2040-2090 (red). Each is normalized by its meridional average (10°S-10°N).

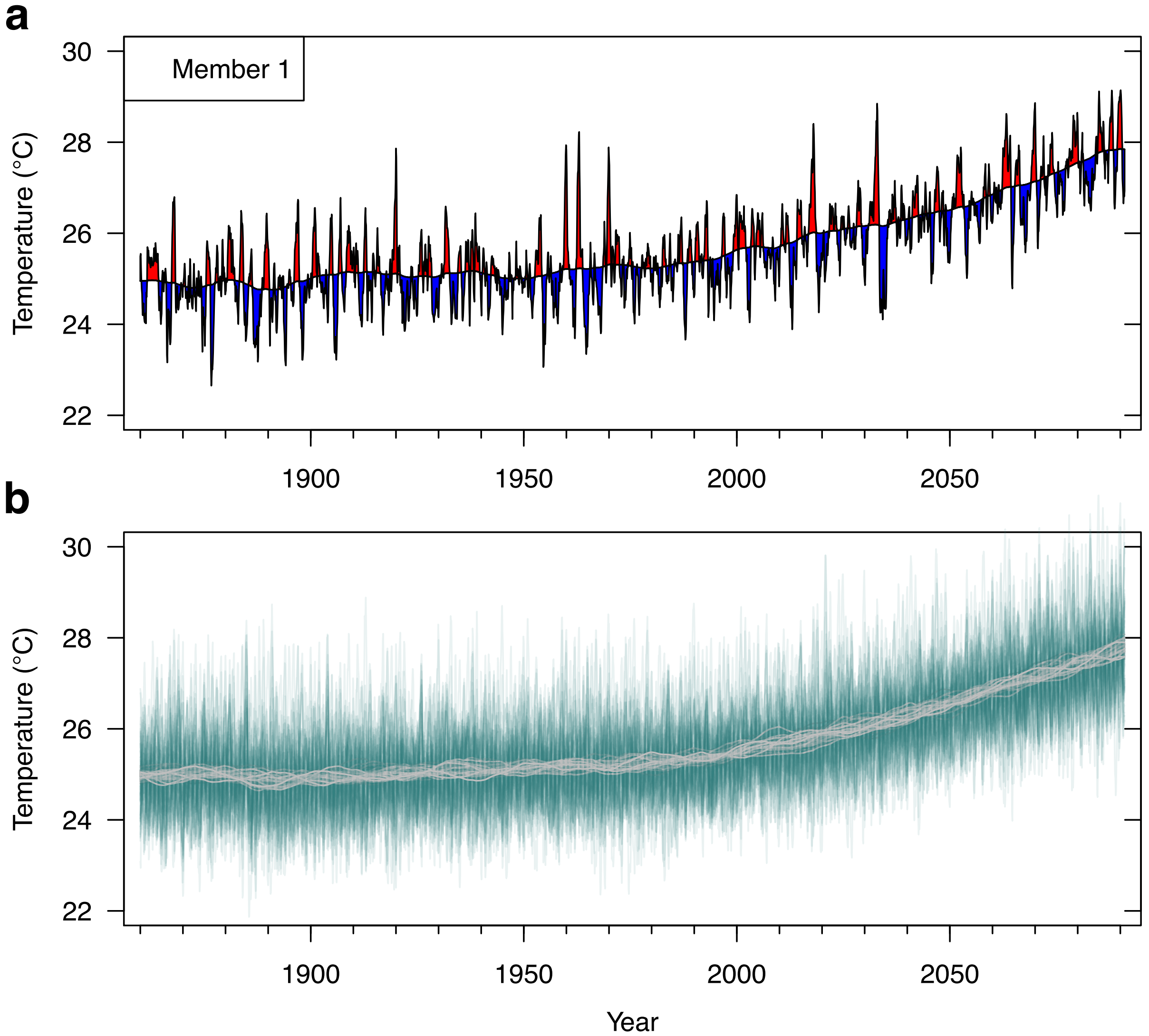

Supplementary Figure S13 | Niño3.4 indices and trends. The Niño3.4 index added to the temperature trend (calculated as a 21-month triangle smoothing) for **a**, the first CESM ensemble member; **b**, the entire CESM ensemble. In **a**, values above the trend are in red, while those below are in blue. In **b**, trends for individual members are shown in grey.

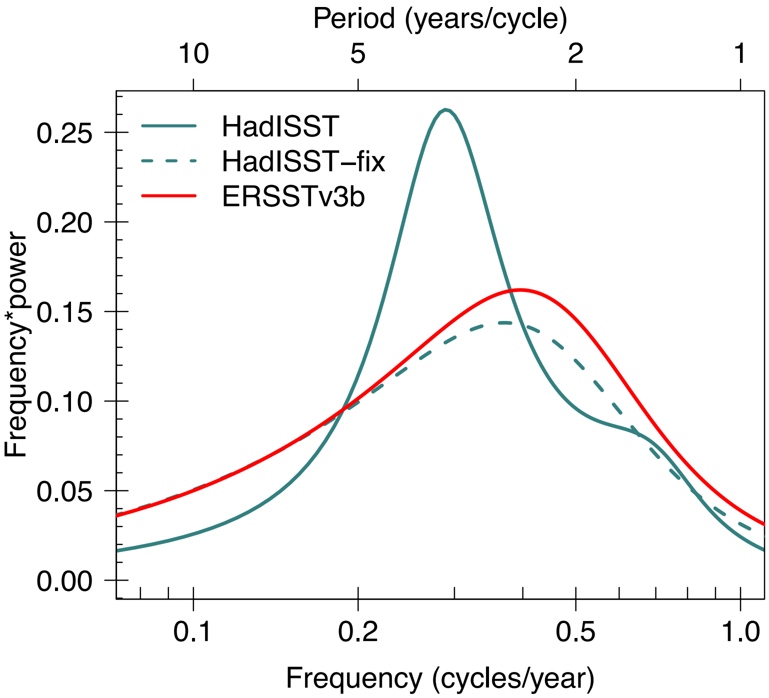

Supplementary Figure S14 | Sensitivity of the maximum entropy power spectrum to its autoregressive fit. 1940-1990 trend-removed Niño3.4 index maximum entropy power spectra for HadISST v1.1 (solid blue-green) and ERSST v3b (red). The HadISST v1.1 spectrum is based on an order-21 autoregressive fit to the time series, while ERSST v3b is based on an order-5 fit. The dashed line shows HadISST v1.1 with an order-5 fit.

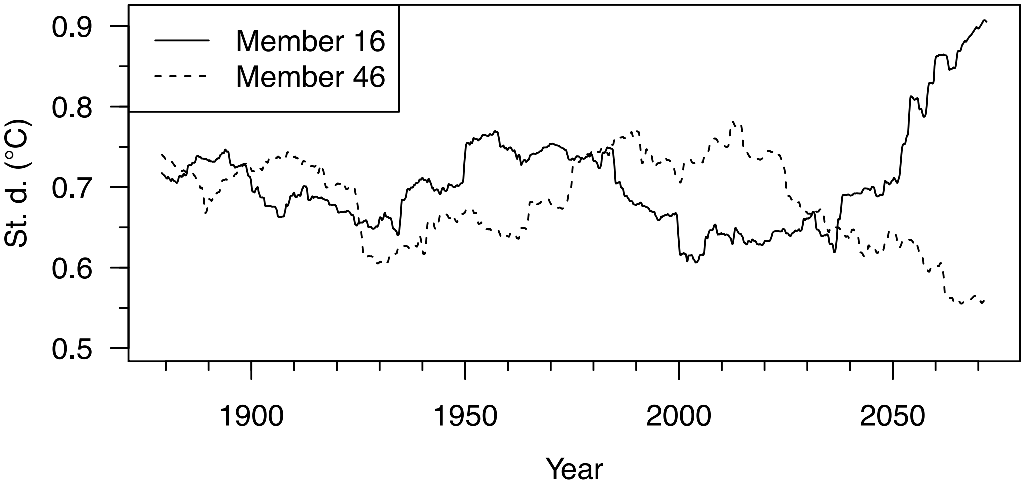

Supplementary Figure S15 | 50-year running standard deviation of the trend-removed Niño 3.4 index for two sample ensemble members. These members are chosen to highlight the different 21st century trends possible under the same forcing. They have the largest positive and negative 21st century trends, respectively.

Supplementary tables

	Control mean	2040-2090 mean	|Control-2040-2090|	p-value	95% Detection sensitivity	95% Detection sensitivity (%)
St. dev. (°C)	0.701	0.698	0.003	0.823	0.026	3.7
Skewness	0.263	0.325	0.058	0.308	0.12	45.9
El Niño count	11.14	11.28	0.14	0.732	0.81	7.3
La Niña count	12.54	12.82	0.28	0.516	0.85	6.8
Extreme El Niño count	6.34	6.28	0.06	0.837	0.58	9.1
EMI st. dev. (°C)	0.394	0.353	0.041	$2.7*10^{-12}$	0.010	2.7

Supplementary Table 1 | Welch's two-sample t-test results comparing ENSO properties of the control CESM ensemble and the 2040-2090 ensemble. The p-value gives the two-sided probability of the given difference in means under the null hypothesis of equal means. A p-value less than 0.05 would mean rejection of the null hypothesis with 95% confidence. The 95% detection sensitivity is the approximate difference in means that would be needed to reject the null hypothesis with 95% confidence. The detection sensitivity is calculated assuming 98 degrees of freedom and that the standard deviation of the given property is constant. The fractional detection sensitivity of skewness is much worse than that of the other properties because the fractional distributions of skewness are much larger (main text Fig. 3).

Modeling Center (or Group)	Institute ID	Model Name	Run ID(s)
Commonwealth Scientific and Industrial Research Organization (CSIRO) and Bureau of Meteorology (BOM), Australia	CSIRO-BOM	ACCESS1.0	r1i1p1
		ACCESS1.3	r1i1p1
Beijing Climate Center, China Meteorological Administration	BCC	BCC-CSM1.1	r1i1p1
Canadian Centre for Climate Modelling and Analysis	CCCMA	CanESM2	r1i1p1
National Center for Atmospheric Research	NCAR	CCSM4	r1i1p1
Community Earth System Model Contributors	NSF-DOE-NCAR	CESM1(BGC)	r1i1p1
		CESM1(CAM5)	r1i1p1
Centro Euro-Mediterraneo per I Cambiamenti Climatici	CMCC	CMCC-CM	r1i1p1
		CMCC-CMS	r1i1p1
Centre National de Recherches Météorologiques / Centre Européen de Recherche et Formation Avancée en Calcul Scientifique	CNRM-CERFACS	CNRM-CM5	r1i1p1
Commonwealth Scientific and Industrial Research Organization in collaboration with Queensland Climate Change Centre of Excellence	CSIRO-QCCCE	CSIRO-Mk3.6.0	r1i1p1
EC-EARTH consortium	EC-EARTH	EC-EARTH	r8i1p1
The First Institute of Oceanography, SOA, China	FIO	FIO-ESM	r1i1p1
NOAA Geophysical Fluid Dynamics Laboratory	NOAA GFDL	GFDL-CM3	r1i1p1
		GFDL-ESM2G	r1i1p1
		GFDL-ESM2M	r1i1p1
NASA Goddard Institute for Space Studies	NASA GISS	GISS-E2-H	r1i1p1-3
		GISS-E2-R	r1i1p1-3
National Institute of Meteorological Research/Korea Meteorological Administration	NIMR/KMA	HadGEM2-AO	r1i1p1
Met Office Hadley Centre (additional HadGEM2-ES realizations contributed by Instituto Nacional de Pesquisas Espaciais)	MOHC (additional realizations by INPE)	HadGEM2-CC	r1i1p1
		HadGEM2-ES	r2i1p1
Institute for Numerical Mathematics	INM	INM-CM4	r1i1p1
Institut Pierre-Simon Laplace	IPSL	IPSL-CM5A-LR	r1i1p1
		IPSL-CM5A-MR	r1i1p1
		IPSL-CM5B-LR	r1i1p1
Atmosphere and Ocean Research Institute (The University of Tokyo), National Institute for Environmental Studies, and Japan Agency for Marine-Earth Science and Technology	MIROC	MIROC5	r1i1p1
Max-Planck-Institut für Meteorologie (Max Planck Institute for Meteorology)	MPI-M	MPI-ESM-MR	r1i1p1
		MPI-ESM-LR	r1i1p1
Meteorological Research Institute	MRI	MRI-CGCM3	r1i1p1
Norwegian Climate Centre	NCC	NorESM1-M	r1i1p1
		NorESM1-ME	r1i1p1

Supplementary Table 2 | Models included in CMIP5 comparison ensemble

Supplementary text

Further verification of low-resolution CESM ENSO.

It is reassuring that the amplitude and periodicity of ENSO in low-resolution CESM are comparable with observations[26] (Figs. 2, 3 in the main text). However, ENSO is dependent on a series of coupled oceanic and atmospheric feedbacks. Error compensation among these feedbacks can lead to an apparently realistic ENSO and projection overconfidence[12,19]. Here we present several additional verifications of the modeled ENSO.

The tropical Pacific mean state, affected by the same feedbacks governing ENSO, can be used as an indicator of a model's ability to capture ENSO[12]. Low-resolution CESM captures the zonal asymmetry of tropical Pacific seasonality (Supplementary Fig. S1). However, notable biases include an offset in the seasonality by about one month and an unrealistic cooling in the western Pacific during April and May.

ENSO variability is observed to peak from November-January. About half of all CMIP3 and CMIP5 models share this peak. Those that capture this phase locking more accurately capture the temperature-dependence of eastern Pacific convective regimes[12], which is an important ENSO feedback[51]. Low-resolution CESM's ENSO shares the observed phase locking (Supplementary Fig. S2). However, model variability is lower in all months except for an unrealistic bump in July and August.

ENSO can be partially understood as a recharge-discharge of equatorial ocean heat content[28]. After a build-up of ocean heat, an El Niño event then releases that heat. The 20°C isotherm depth can be used as an indicator of ocean heat content. The cross-correlation of eastern equatorial Pacific SST and equatorial Pacific 20°C isotherm depth allows insight into whether a model can capture the recharge-discharge ENSO mechanism[29]. Observational analysis shows a positive cross-correlation value at negative lags, which means that equatorial ocean heat increases lead increases in eastern Pacific SST[52]. Low-resolution CESM reproduces this peak at negative lags, indicating some recharge-discharge mechanism governing ENSO (Supplementary Fig. S3).

Additional Spectral Analysis.

In the main text, we compare the ensemble ENSO spectra for three forcing regimes/time periods: the unforced/control ensemble, a historical/1940-1990 ensemble, and a projections/2040-2090 ensemble. This comparison is done with a three-panel plot of the spectra and the 5th, 50th, and 95th percentiles of each distribution. Here, we first verify that the distribution of the natural log of normalized power at a given frequency is approximately normal with a quantile-quantile plot (Supplementary Fig. S7). Based on this approximation, we then plot the 95% confidence interval for the mean spectrum of each time period ensemble (Supplementary Fig. S4). For the Niño3.4 region, there is sizeable overlap of the confidence intervals at ENSO frequencies (~2-7 years). This again contrasts with an analysis of the EMI, for which there is a noticeable decrease in power at those frequencies. To highlight the sensitivity of these results to the spectral analysis method, we repeat our analyses with a fast Fourier transform. General results are unaffected (Supplementary Figs. S5, S6).

Additional statistical comparison.

In the main text, we compare the ENSO standard deviation, skewness, and count of El Niño events, La Niña events, and extreme El Niño events in our three time periods/forcing regimes using box-whisker plots. Here we perform a t-test of the hypothesis that the control and 2040-2090 ensembles have equal means (Supplementary Table S1). Before using this t-test, we first verify that the statistical properties have normal distributions using quantile-quantile plots (Supplementary Fig. S7). After confirming approximate normality, we use Welch's two-sample t-test. We find that none of the statistical properties examined for the Niño3.4 region has a two-sided p-value less than 0.05, that is, we cannot reject the null hypothesis of equal means with 95% certainty. Comparing control with 1940-1990 and 1940-1990 with 2040-2090 (not shown in table), there is one p-value less than 0.05: the 1940-1990 vs. 2040-2090 La Niña counts (p-value = 0.044). However, given the 15 t-tests performed, a single p-value this small would not be unlikely when sampling from identical normal distributions. Given this fact and the much larger p-values for other La Niña count comparisons, we do not focus on this result. The null result for the Niño3.4 region contrasts with that for the EMI.

In Supplementary Table S1 we also estimate the difference in the means that would be required to reject the null hypothesis with 95% certainty. This detection sensitivity is calculated assuming the ensemble spread of the given property is constant and that there are 98 degrees of freedom. As an example, the 95% detection sensitivity of the ENSO standard deviation is 3.7%, so we would reject the null hypothesis of equal means with 95% certainty if the change in standard deviation were larger than 3.7%.

Mean state changes and their relationship with ENSO.

Recent analyses of CMIP5 have shown that, while changes in frequency and anomalous temperatures associated with ENSO are uncertain, precipitation-based metrics indicate extreme El Niño and La Niña events become more common under global warming[18,53]. These more frequent extreme events are linked to robust changes in the mean state of the CMIP5 ensemble equatorial Pacific. Under RCP8.5, the CMIP5 ensemble undergoes preferential warming of the eastern equatorial Pacific. The lower east-west and meridional background temperature gradients mean that convection in the eastern equatorial Pacific is more frequently established during El Niño events[18]. The low-resolution CESM ensemble also undergoes preferential warming of the eastern Pacific under RCP8.5 (Supplementary Fig. S11a). Additionally, this preferential warming is correlated with future changes in ENSO variability: the stronger the preferential warming of the East, the more likely ENSO variability increases (Supplementary Fig. S11b). This correlation is consistent with previous studies, which have found that a more active ENSO is associated with warmer east Pacific temperatures[43,54,55].

Index trend removal.

We use a 211-month triangle smoothing of the index time series to calculate a long-term trend. There are some differences in this trend estimate between ensemble members (Supplementary Fig. S13). The trend-removed index loses some year-to-year variability; however, these differences are much smaller than the overall variability of the index. The standard deviation of these trends about their mean is ~0.09°C while the standard deviation of the trend-removed indices about that mean is ~0.68°C.

Trend removal is warranted in order to assess how the variations are changing. Without any trend-removal, the Niño3.4 time series has a strong positive upward trend, especially in the 21st century. Retaining this in the analysis would skew our ENSO statistics: for example, a neutral year (relative to the average state in a ~few year window) in the late 21st century would classify as an El Niño if the index were normalized to 20th century temperatures. In this paper we compare 50-year time periods. If we normalize the 2040-2090 index time series to the average in those years, there is still a sizeable trend within the window. This increases the variance about the windowed mean, even if the average event strength and frequency (again relative to the average state in a ~few year window) do not change.

Sensitivity of the maximum entropy power spectrum to the autoregressive fit

With the maximum entropy technique, the Akaike Information Criterion (AIC) is used to determine the order of an autoregressive fit, which is in turn used to create the power spectrum. The power spectrum is sensitive to the order of the autoregressive fit. For example, the HadISST v1.1 and ERSST v3b trend-removed Niño3.4 indices are nearly identical over 1940-1990. However, applying the maximum entropy technique to these indices, the HadISST v1.1 AIC-determined order is 21, while it is 5 for ERSST v3b. The resulting spectra are notably different (Supplementary Fig. S14). To verify that the difference between the two spectra is due to their autoregressive fits, we redo the fit and spectrum estimate for HadISST v1.1, forcing the autoregressive fit to the same order as for ERSST v3b. With this adjustment, the difference between the two spectra is much smaller. We note that while this sensitivity of the maximum entropy technique can be considered a weakness, the basic results of our spectral analysis are unaffected using a fast Fourier transform (Supplementary Figs. S5, S6).

Supplemental References

51. Lloyd, J., Guilyardi, E., & Weller, H. The role of atmosphere feedbacks during ENSO in the CMIP3 models, Part III: the shortwave feedback. *J. Clim.* **25,** 4275–4293 (2012).

52. Meinen, C. S., & McPhaden, M.J. Observations of warm water volume changes in the equatorial Pacific and their relationship to El Niño and La Niña. *J. Clim.* **13,** 3551–3559 (2000).

53. Cai, W. *et al.* Increased frequency of extreme La Niña events under greenhouse warming. *Nat. Clim. Change* **5,** 132-137 (2015).

54. Ogata, T., S.-P. Xie, A. Wittenberg, and D.-Z. Sun, Interdecadal amplitude modulation of El Niño/Southern Oscillation and its impacts on tropical Pacific decadal variability. *J. Clim.* **26**, 7280-7297 (2013).

55. Wittenberg, A. T. Low-frequency variations of ENSO. *U.S. CLIVAR Variations* **13** (1), 26-31 (2015).
